# Portable Ultrasound Research System for Use in Automated Bladder Monitoring with Machine-Learning-Based Segmentation

**DOI:** 10.3390/s21196481

**Published:** 2021-09-28

**Authors:** Marc Fournelle, Tobias Grün, Daniel Speicher, Steffen Weber, Mehmet Yilmaz, Dominik Schoeb, Arkadiusz Miernik, Gerd Reis, Steffen Tretbar, Holger Hewener

**Affiliations:** 1 Department of Ultrasound, Fraunhofer Institute for Biomedical Engineering, 66280 Sulzbach, Germany; tobias.gruen@ibmt.fraunhofer.de (T.G.); daniel.speicher@ibmt.fraunhofer.de (D.S.); steffen.weber@ibmt.fraunhofer.de (S.W.); steffen.tretbar@ibmt.fraunhofer.de (S.T.); holger.hewener@ibmt.fraunhofer.de (H.H.); 2 Department of Urology, Faculty of Medicine, Universitätsklinikum Freiburg University of Freiburg, Hugstetter Str. 55, 79106 Freiburg, Germany; mehmet.yilmaz@uniklinik-freiburg.de (M.Y.); dominik.stefan.schoeb@uniklinik-freiburg.de (D.S.); arkadiusz.miernik@uniklinik-freiburg.de (A.M.); 3 DFKI—German Research Center for Artificial Intelligence, Trippstadter Straße 122, 67663 Kaiserslautern, Germany; reis@dfki.de

**Keywords:** POCUS, multichannel system, channel data, bladder monitoring, POUR, machine-learning, segmentation

## Abstract

We developed a new mobile ultrasound device for long-term and automated bladder monitoring without user interaction consisting of 32 transmit and receive electronics as well as a 32-element phased array 3 MHz transducer. The device architecture is based on data digitization and rapid transfer to a consumer electronics device (e.g., a tablet) for signal reconstruction (e.g., by means of plane wave compounding algorithms) and further image processing. All reconstruction algorithms are implemented in the GPU, allowing real-time reconstruction and imaging. The system and the beamforming algorithms were evaluated with respect to the imaging performance on standard sonographical phantoms (CIRS multipurpose ultrasound phantom) by analyzing the resolution, the SNR and the CNR. Furthermore, ML-based segmentation algorithms were developed and assessed with respect to their ability to reliably segment human bladders with different filling levels. A corresponding CNN was trained with 253 B-mode data sets and 20 B-mode images were evaluated. The quantitative and qualitative results of the bladder segmentation are presented and compared to the ground truth obtained by manual segmentation.

## 1. Introduction

Ultrasound imaging is a frequently used method for postoperative monitoring of the urinary bladder. Depending on the surgical context, different clinical conditions that need close monitoring can occur. Post-operative urinary retention (POUR) is a frequent problem for various reasons (e.g., intravesical blood clotting) that can lead to bladder overdistension and needs rapid detection and medical intervention. On the other hand, invasive procedures such as catheterization present significant discomfort for patients and can lead to infections or even trauma of the urinary tract. In contrast, ultrasound imaging is fully non-invasive and has already shown its potential for bladder monitoring [1,2,3]. Accurate bladder volumes can be extracted from 3D ultrasound data; however, reliable qualitative information about potential bladder overdistension can already be derived from 2D B-mode (brightness mode) ultrasound images.

In order to efficiently prevent POUR and directly initiate therapeutic measures if an increased amount of urine or blood is detected in the urinary bladder, the bladder should be monitored at frequent intervals, which is not possible in a clinical environment where ultrasound investigations are mostly performed using standard sonography equipment based on hand-held probes. Accordingly, when defining a tool for ideal postoperative follow-up and the prevention of related complications in the 24–48 h period after surgery, different challenges and requirements arise. First, the system must be portable, such that the mobility of the patient is ensured. Second, the probe must be self-adhesive or pad-like (in contrast to hand-held probes that require the presence of a sonographer). Third, (image or signal) data must be automatically analyzed to retrieve diagnostic features that are relevant for the identification of a potential complication (e.g., a bladder volume above a defined threshold in the context of POUR monitoring or specific scattering properties as a result of blood clots in the bladder). In a research context, where the optimal signal and image processing still needs to be defined, this results in a need for RF or even better pre-beamformed channel data access. In particular, the third requirement allows the use of analysis methods beyond pure image-based segmentation and classification. We recently showed in other applications that machine learning approaches can be applied to raw radio-frequent ultrasound data prior to image formation for classification tasks with a high accuracy [4]. Radio-frequent data with a high dynamic range (16-bit amplitude quantization) and ultrasonic wave phase information at high digitalization rates of up to 50 MHz contain a lot more informational content than scan-converted ultrasound images. During scan conversion, typically more than 90% of the raw ultrasound wave information is lost during image formation and cannot be used in image-based processing.

To the best of our knowledge, there are no systems available that fulfil the above defined requirements. The use of advanced classification approaches is not possible with classical clinical sonography systems, as they do not provide access to radio frequent ultrasound data. Ultrasound systems for research applications such as the Vantage Ultrasound System (Verasonics, Inc. Redmond, WA, USA), the ULA-OP [5], the systems from the Technical University of Denmark [6] or the DiPhAS by Fraunhofer IBMT (Sulzbach, Germany) [7] provide access to this type of data, but are mostly not certified for clinical use, and more importantly, they are complex, bulky and costly devices. The latest generation of point of care ultrasound (POCUS) devices, such as the Butterfly iQ or Vscan [8] by GE has decreased the costs by an order of magnitude when compared to high-end sonography machines, and can be used in bedside settings due to their miniaturization. However, the availability of care staff still represents a limiting factor when it comes to frequent monitoring postoperatively. Finally, dedicated devices for bladder monitoring such as DFree (Triple W, Tokyo, Japan) or SENS-U [9] (Novioscan, Nijmegen, The Netherlands) have a particular focus on incontinence management. These systems directly generate bladder filling level-related parameters and do not provide access to the underlying ultrasound signals. Other ultrasound systems optimized for urological applications such as BladderScan (Verathon, WA, USA) measure the bladder volume, but are based on hand-held probes, which limits their suitability for continuous monitoring.

In summary, all these devices optimized for the daily clinical routine (or for home-care settings in the case of DFree or SENS-U) are difficult to utilize in research applications, where customized signal and image processing algorithms need to be applied to the data. In particular, machine-learning based approaches have been shown to have tremendous potential for automated segmentation of ultrasound data [10], and have been reported in particular for breast imaging [11], coronary arteries [12], and thyroid [13] or different tumors [14]. In comparison to these applications, where the anatomy is more complex and the contrast difference is reduced, bladder segmentation represents an ideal use case for ML-based approaches due to the low echogenity and the resulting high contrast to surrounding tissue. Multiple ultrasound imaging devices, including mobile ones like Butterfly iQ+ (Butterfly Network Inc, Guilford, CT, USA), already include automated bladder segmentation and volume estimation, but the shape of the hand-held transducer does not allow long-time monitoring as a wearable.

In light of the somewhat contrary requirements of an ideal urinary bladder monitoring system that also provides full data access, and thereby can be flexibly used in research applications, we developed a new portable ultrasound system (mobile ultrasound equipment—MoUsE). Despite being validated in this first application in the context of bladder monitoring, the MoUsE can also be used as a general-purpose ultrasound research system since full access to the transmit and receive pipeline is provided.

## 2. Materials and Methods

### 2.1. Portable Multichannel Electronics with Research Interface

**Figure 1:**
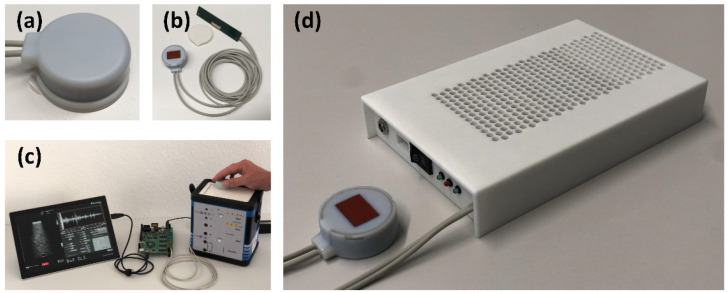
MoUsE system overview with close up of 32 element transducer housing (**a**), transducer with cable, custom connector PCB and disposable patch (**b**), MoUsE PCB tested on phantoms prior to integration (**c**), and the final system integrated with passive cooling in a 3D printed housing (**d**).

The MoUsE is a compact ultrasound system integrated into a 3D printed housing (Figure 1) with dimensions of 184 mm × 123 mm × 33 mm and a total weight of 610 g, thus ensuring its portability. It is driven by a 12V medical power supply which can be replaced by lithium-ion battery packs for future fully mobile applications. Detailed specifications are given in Table 1. All system functionalities, including generation of transmit signals, amplification and digitization of receive signals, storage and communication (via USB 3.0) to a PC/tablet controlling the device are implemented on the same main printed circuit board (PCB). Data management, communication and sequence control are handled in the integrated ZYNQ-7 FPGA. An on-board low voltage (LV) power supply generates the required power levels for the logic components.

A compact high voltage (HV) power supply that generates the ± 100 V of transmit voltage for each of the octal (8-channel) transmit receive ICs was implemented on a second PCB mounted on the main PCB. A frequency range of 100 kHz–10 MHz was defined as the transmit bandwidth.

In principle, transmit signals can be freely defined within the limits of the tri-state programmable ICs, for instance, using pulse width modulation (PWM); however, only rectangular bursts with adjustable length and frequency have been implemented in the software so far. The internal system clock of 160 MHz is used for the definition of the transmit signals. Receive signals are digitized with up to 50 MSa/s with a resolution of 12 bit and are transferred as pre-beamformed channel data via USB 3.0 to a PC/tablet for image reconstruction. The receive data can be amplified by up to 44.3 dB with different linear or customized TGC settings. No analog preprocessing is performed on-board beyond bandpass filtering and (optional) data accumulation (corresponding to averaging) for improvement of the signal to noise ratio (SNR). Interfaces for wireless (IEEE 802.11 b/g/n (1 × 1)) communication and the transfer of pre-beamformed channel data are foreseen in the hardware design but not yet implemented. The system uses a sleep mode to switch off the transceiver ICs for stand-by between long-term measurements to reduce power consumption.

### 2.2. Transducer Design and Manufacturing

**Figure 2:**
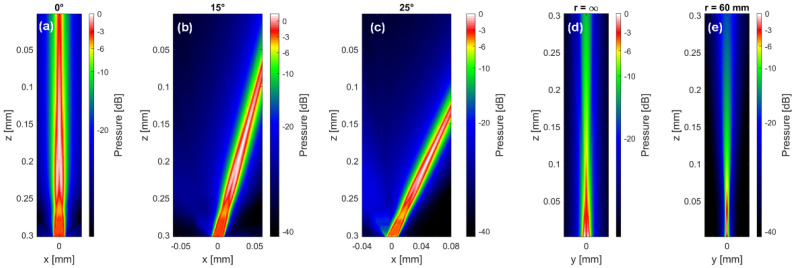
Acoustic pressure distribution simulation performed during specification phase of the MoUsE transducer. (**a**–**c**) Plane wave transmission under different angles to investigate the steering capabilities and the identify potential grating lobes, (**d**,**e**) single element elevational sound field without focusing (**d**) and with lens focusing to 60 mm (**e**).

The MoUsE can be driven with all kinds of 32-element transducers using the given pinout or via transducer connection adapter. However, in the context of the first application being used for automated bladder monitoring, a 32-element phased array transducer was developed. The transducer properties were defined in a sound field simulation study using the in-house developed sound field simulation software tool SCALP based on point source synthesis (Figure 2). A pitch of 500 µm with a kerf of 50 µm and element sub-dicing were chosen as a compromise between sensitivity (profiting from larger element size) and beam steering capabilities (decreasing with larger element size). To improve the elevational resolution, a focusing silicon lens was applied to the element of elevational size of 11.5 mm. The array was manufactured from a soft PZT material (3203 HD), the center frequency was adjusted to 3 MHz and two matching layers were applied for improved bandwidth. Connection to the MoUsE electronics was achieved by two 16-core micro-coax cables directly soldered to the customized connector PCB, which was preferred over a solution involving a commercial connector for the sake of compactness. The acoustic block was finally integrated into a 3D printed cylindrical housing of 40 mm in diameter and a height of 17.5 mm. For long-term monitoring applications, a fixation concept involving an acoustically transparent adhesive tape could optionally be used.

### 2.3. Beamforming and Software

**Figure 3:**
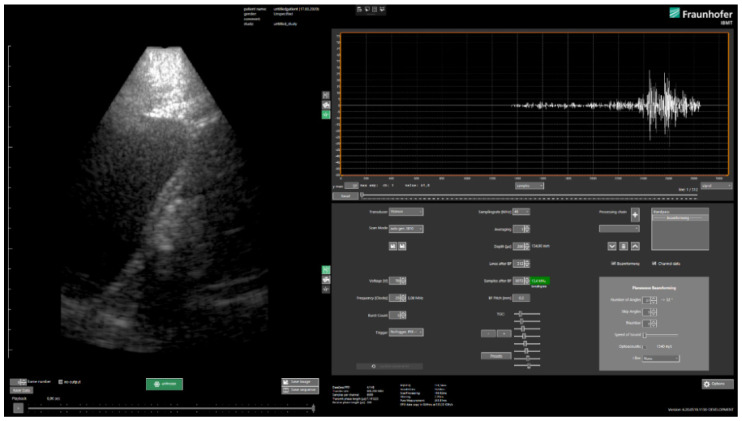
Clinical style user interface USPilot.

Image reconstruction is performed in real-time using a GPU (OpenCL, Khronos Group, Beaverton, OR, USA)-based implementation of plane wave compounding [15] approach in the in-house developed clinical style user interface USPilot (Figure 3). Other reconstruction methods can easily be implemented via an SDK. The number of plane wave angles, as well as the increment can be freely selected by the user. Other transmit parameters such as the frequency, the burst count or the voltage can be adjusted as well. On the receive side, the data sampling rate, averaging factor and TGC can be selected.

The reconstruction can be adjusted in terms of the size and resolution of the reconstruction grid (lateral and axial pixel/sample count), the speed of sound and apodization. Furthermore, customized algorithms (e.g., bandpass filtering or alternative beamforming approaches) can be inserted into the (real-time) reconstruction pipeline. The software allows the visualization of reconstructed (compounded) B-scan images as well as the pre-beamformed channel data (in time or frequency) domain, which makes it ideal not only for clinical research, but also for educational purposes or research on reconstruction algorithms. In addition to controlling the system via the USPilot, an open programming interface (C#/C++/Matlab with SDK) is made available, which provides access to the same transmit, receive and beamforming parameters as in the case of the UI. A custom but open binary data format (*.orb) is chosen for storage of the pre-beamformed and reconstructed ultrasound data. Meta-data such as transmit and receive parameters are stored with the actual ultrasound data by default and import tools for Matlab/Python/C/C++ are made available.

### 2.4. ML-Based Segmentation Algorithm

**Figure 4:**
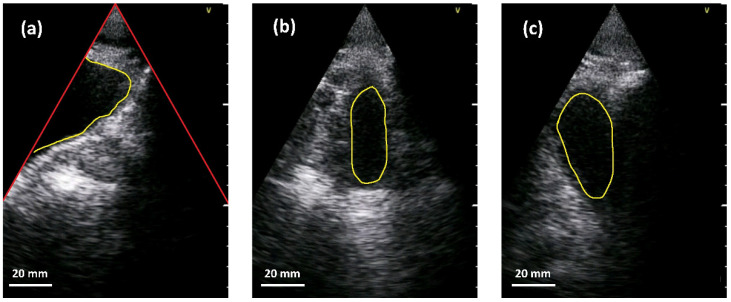
Examples of bladder segmentation (yellow). Red lines in the left sub-image indicate the borders of the ultrasound fan above which there is no valid information. (**a**) Partially visible bladder. (**b**) Almost empty bladder near the center of the US-fan. (**c**) Additional dark regions due to pubic bone shadow or colon.

We trained a neural network to segment the bladder into abdominal ultrasound images and encountered two main challenges when implementing the network. On the one hand, the limited space and computational resources available at inference time and on the other hand, the quality of abdominal ultrasound images can be very challenging. Figure 4 shows an example: in the left sub-figure, a (partially filled) bladder appears mainly as a dark region in the image since little sound is reflected by the fluid. In addition, the bladder is only partially imaged and merges seamlessly into the black area outside the ultrasound fan. This situation is usually the case in corpulent patients. As can be seen in the upper part of the segment, weak echoes might occur in cases where the side lobes of the ultrasound beam intersect with the bladder tissue. A very different situation is shown in the middle sub-figure. Here, the (almost empty) bladder is located in the middle of the ultrasound fan. Lastly, the right sub-figure depicts a situation where other anatomical structures, e.g., the pubic bone or the colon, generate a large dark region that might fuse with the bladder. Please note that the appearance of different anatomical parts can be very similar in the images.

We started development using a Mask-R-CNN architecture [16,17] to segment the bladder. However, we found that the model size of approximately 0.5 GByte was way too large for the intended purpose. A second drawback was that the network tended to overfit to the data, since only very few images (253) were available for training. We therefore decided to use a U-Net architecture [18] in a minimal configuration. We set the network up to compute a 2-class segmentation (bladder, non-bladder). The original ultrasound images (1056 × 720) were down-sampled to a resolution of 528 × 352 and reduced to a single color channel. In total, we acquired 253 data sets (each consisting of one B-mode image), which were acquired from 20 human volunteers as training data for the CNN. For both the contracting and expanding paths, we used 5 successive blocks. We started with 6 channels for the first layer and doubled the channel number with each successive layer, resulting in a total of 96 channels at the bottleneck. For expansion we used up-sampling followed by convolution. Training was performed using a batch size of 4 with a learning rate of 0.00002. Using these parameters, the network converged within 400 epochs. The resulting network was less prone to overfitting than the original attempt. We found however that the amount of data was still too low. More importantly, we found that the network had issues in detecting the virtual border of a scan in the image. In particular, if the ultrasound response for a partially imaged bladder was very weak, the resulting segment often extended into the illegal region of the image, i.e., outside the ultrasound fan. Additionally, we often found cases where other dark regions were segmented as bladder.

To this end, we extended the dataset by re-sampling the images so that one of the fan sides coincided with one of the image borders. Furthermore, we flipped images and ground truth on the vertical as well as the horizontal axis. This way we increased the number of images by a factor of 20. Training the network using the augmented data effectively prevented overfitting. Furthermore, and probably much more importantly, the network learned how artifacts and the bladder differ.

For the trained network, we computed an IoU above 0.75 but below 0.9 for all images. We checked segmentations and ground truth and found, interestingly, that the computed segmentations were consistently tighter (smaller) than the ground truth provided by medical experts. The ground truth segmentation was performed by one experienced urologist using the VIA annotation tool [19]. A second experienced urologist performed the validation of the ground truth. Consulting with the experts revealed that the network only segmented the interior of the bladder while the experts partially included the bladder tissue. This unintended result proved to be beneficial for the application at hand. Since we want to estimate the bladder volume, including the tissue would lead to a systematic error that, in particular, depends on the volume itself.

We are working on a further reduction in the network size. The original network size was 340 MBytes. We were able to reduce its size with various pruning strategies [20] to under 300 MBytes without significantly sacrificing the quality of the results. This size is still too large to be run efficiently on a mobile device. Additionally, the inference times need to be decreased significantly. Currently, the network does inference on the target device at approximately 6.4 s per frame. Although this would be more than sufficient for a regular check of the bladder volume, the system would not be able to perform any other tasks in the meanwhile. In the use-case of regular checks of the bladder volume and content, such an inference frame rate might still be acceptable for long-time monitoring. The integration of such a model in the processing pipeline will be implemented by supporting the ONNX model format with the C# runtime using Microsoft ML.NET in the future.

## 3. Results

### 3.1. Characterization of Electronics

**Figure 5:**
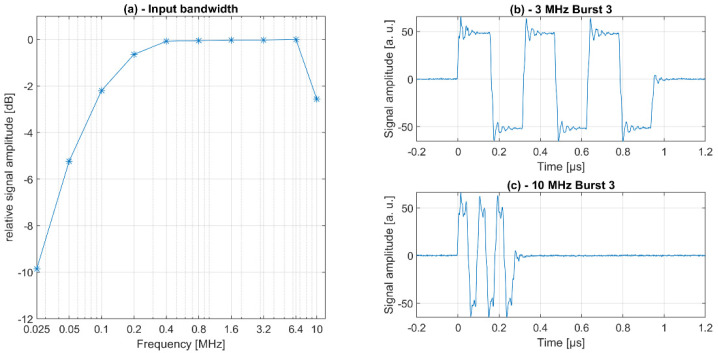
Assessment of MoUsE electronics input bandwidth (**a**) and signal fidelity (**b**,**c**) as a function of frequency.

The transmit and receive paths of the electronics were characterized with respect to the bandwidth. First, for the assessment of the transmit bandwidth, an 80 mVpp sinus signal of varying frequency from a signal generator was digitized by the electronics and the amplitude of the digitized signal was characterized (Figure 5a). As can be seen, the input bandwidths significantly decrease below 100 kHz and above 10 MHz. Furthermore, we evaluated the signal fidelity by generating rectangular bursts of 3 cycles at different frequencies (Figure 5b,c). The electrical signals were measured on the connector PCB with an oscilloscope and minor overshooting was observed.

### 3.2. Transducer Characterization

**Figure 6:**
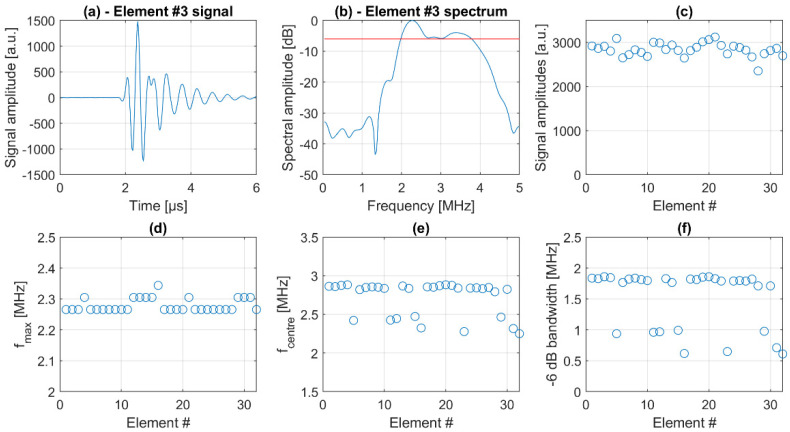
Analysis of transducer performance by charaterization of pulse-echo data from a steel reflector. Single element signals in time (**a**) and frequency (**b**) domain, where the red line depicts the −6 dB threshold. Element sensitivity statistics (**c**), maximum frequency, center frequency and bandwidth statistics (**d**–**f**).

For the assessment of the transducer performance, echo signals from a steel reflector generated by excitation of individual transducer elements with a rectangular burst 1 were evaluated. Figure 6a shows a typical time domain echo signal of one of the transducer elements with the corresponding spectrum in Figure 6b. Each of the signals was analyzed with respect to the maximum signal amplitude in order to compare the transmit-receive sensitivity of the transducer elements. As can be seen in Figure 6c, the element sensitivity is very homogeneous with a relative standard deviation of only 5.6%.

For all elements, the maximum frequency is around 2.3 MHz with a standard deviation of 1% (Figure 6d). The center frequency and the −6 dB bandwidth seem to vary more strongly (Figure 6e,f); however, this is an artifact due to a frequency dip around 3 MHz just below the −6 dB line in the spectrum (red line in Figure 6b). If we neglect this minor dip, the average center frequency of the transducer is 2.9 MHz with a −6 dB bandwidth of approximately 60%.

### 3.3. System Characterization/Standards

In view of using the system on probands in the context of an exploratory clinical study, the system’s compliance with respect to medical device standards was verified by certified laboratories. In particular, the acoustic output was characterized according to IEC 60601-2-37, where the maximum pressure, the mechanical and thermal index as well as the intensity were assessed. All parameters remain well below the threshold for diagnostic ultrasound (e.g., MI < 0.5 and I_SPTA_ < 5 mW/cm²). Furthermore, the electrical safety was tested according to IEC 60601-1 and the electromagnetic compatibility (e.g., immunity and emission) was tested according to IEC 60601-1-2. The system complied with the standards in both tests.

### 3.4. Imaging Performance

#### 3.4.1. Reconstruction Speed

When considering the achievable reconstruction speed and the system frame rate, the data transfer from the electronics to the PC/tablet, where the GPU-based reconstruction is implemented, represents a bottleneck, rather than the reconstruction itself. With the used setup (Surface Pro 7 with Intel Core i7-7660U, 16GB RAM, Intel Iris Plus Graphic 640, Microsoft, Redmond, WA, USA), up to 300 frames of pre-beamformed channel data could be transferred when a sampling rate of 40 MSa/s and an image depth of 8 cm were chosen. Both parameters have a direct impact on the number of transferred frames per second; however, this is not totally linear due to some communication overhead. Since less time is needed for GPU-reconstruction than for data transfer, plane wave imaging can be performed with 300 frames/s for the above-described parameters with 23 B-scans per second and using compounding with 13 angles.

#### 3.4.2. Resolution

The image resolution was characterized using wires with a diameter of 150 µm in a water tank at different depths. Pre-beamformed channel data were acquired after transmitting 21 plane waves in an angle range of ± 16°. Reconstruction was performed offline in Matlab (The MathWorks, Inc., Natick, MA, USA) with the highest resolution to allow better assessment of the lateral extent of the point spread function (PSF).

The FWHM (Full Width Half Maximum) was characterized as a function of depth (wires in distances between 1 cm and 10 cm from the transducer aperture) and as a function of the number of compounding angles (from 1 to 21). Furthermore, different beamforming approaches were investigated from conventional delay and sum (DAS) to coherence beamforming (COH) [21,22] or non-linear filter approaches based on signal statistics (STD) [23].

**Figure 7:**
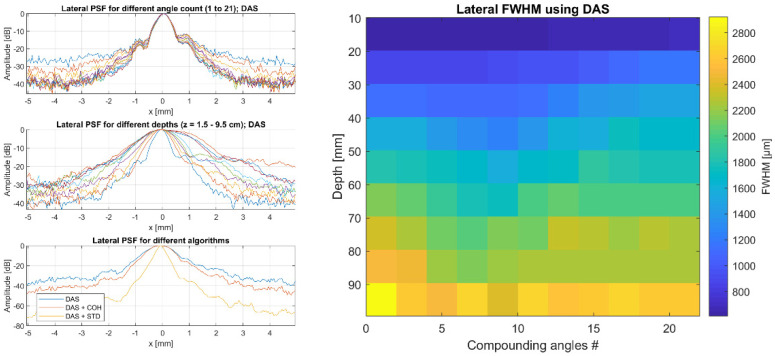
Lateral PSF of the MoUsE system equipped with our 32-element 3 MHz phased array probe. (**Left**) FWHM as a function of plane wave compounding angle count for a constant depth, as a function of depth for constant plane wave compounding angle count and FWHM obtained with different reconstruction approaches. (**Right**) 2D plot of FWHM as a function of angle count and depth for conventional DAS beamforming.

The lateral FWHM ranges between 300–800 µm depending on the chosen algorithm for the targets closest to the aperture and between 1300–2800 µm for those that are 10 cm away. In all cases, the STD reconstruction significantly improves the lateral resolution when compared with simple DAS. Furthermore, Figure 7 shows that increasing the number of compounding angles does not always lead to an improved resolution. In fact, depending on the depth, an ideal resolution is achieved with 5–10 compounding angles. This can be explained by trailing wave artifacts, which are not taken into account in the DAS beamforming.

#### 3.4.3. Signal to Noise Ratio

**Figure 8:**
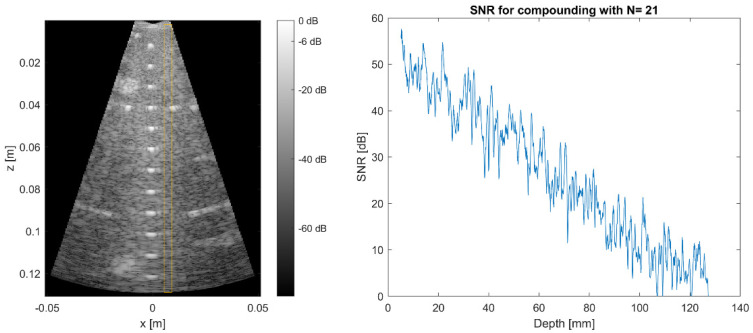
B-mode image acquired with 21 compounding angles used for calculation of the SNR (**left**). Depth-dependent SNR of compounded data (**right**).

The depth-dependent system’s *SNR* was characterized using data from a CIRS multipurpose phantom. One hundred consecutive image acquisitions were performed with the CIRS phantom in the same position and the reconstructed, compounded and enveloped filtered data were analyzed (prior to logarithmic compression). Each depth mean values *µ* and standard deviation values *σ* along a central image line in the yellow frame in Figure 8 were used to calculate the depth-dependent *SNR* as suggested in [24].


(1) $$SNR\left(z\right)=20\cdot log_{10}\left(\mu \left(z\right)/\sigma \left(z\right)\right)$$

To achieve the ideal *SNR*, the data were acquired in a compounding mode with 21 angles in the range of ± 16°. Conventional delays-and-sum beamforming without additional contrast-enhancing filter was used to reconstruct the data.

#### 3.4.4. Contrast

Assessment of the image contrast was performed by scanning lesions in a standard ultrasound imaging phantom (CIRS multipurpose phantom Model 040GSE, CIRS, Norfolk, VA, USA). The contrast ratio (*CR*) and the contrast to noise ratio (*CNR*) as defined in [25] were taken as metrics for quantification of the image contrast behavior:


(2) $$CR=20\cdot log_{10}\left(\mu_{lesion}/\mu_{bck}\right)$$

(3) $$CNR=\frac{\left|\mu_{bck}-\mu_{lesion}\right|}{\sqrt{\sigma_{bck}{}^{2}-\sigma_{lesion}{}^{2}}}$$

**Figure 9:**
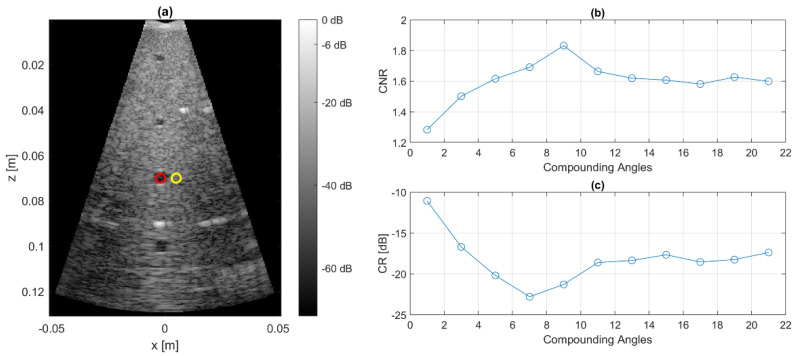
B-mode image of CIRS phantom (**a**) taken for assessment of CNR and CR by analysis of lesion (red ROI) and background (yellow ROI). The x-dimension is in the lateral dimension of the ultrasound array and the z-dimension is in the axial direction (ultrasound propagation direction). CNR and CR (**b**,**c**) are calculated as metrics based on the mean values and standard deviations inside the ROIs.

Plane wave compounding data were acquired with a varying number of angles between 1 and 21. The metrics were then assessed as a function of the number of compounding angles. For this purpose, the mean values *µ* and the standard deviation *σ* inside defined image regions (red circle: lesion; yellow circle: background in Figure 9a) were calculated.

### 3.5. Segmentation

**Figure 10:**
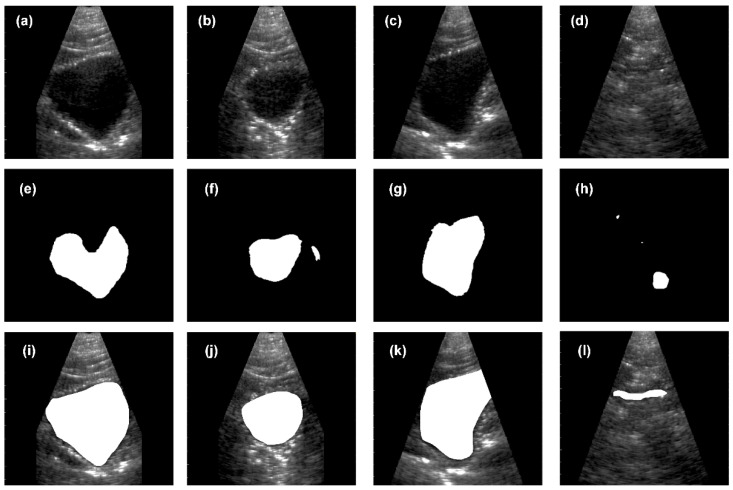
First investigation of the combination of ultrasound B-mode images acquired with the MoUsE system and the described CNN for automated segmentation of human bladder. (**a**–**d**) Ultrasound B-mode data (plane wave compounding, 21 angles), (**e**–**h**) segmentation results from the CNN, and (**i**–**l**) ground truth segmentation (performed manually by an experienced urologist).

To validate the quality of the trained CNN, ultrasound B-mode images from human bladders with different filling levels were acquired from four male volunteers with the MoUsE system. In this first study, 20 data sets (each consisting of one reconstructed B-mode image) were collected. None of these data sets is included in the 253 data sets used for training of the CNN. For image acquisition, an ideal position for the probe on the abdomen was identified based on the real-time feedback of the MoUsE system. Images of the bladder at different filling levels were then acquired with the probe at this position. When it comes to the beamforming approach, plane wave compounding with 21 angles was chosen. Examples of different bladder images can be seen in Figure 10a–d. In a second step, the images were automatically segmented using the above-described CNN. Examples of the segmentation for four different data sets are given in Figure 10e–h, where different situations can be identified. In Figure 10e, the upper part of the bladder, which is closest to the probe, is not identified as part of the bladder by the CNN. This might be due to clutter signals in this part of the image. In Figure 10f, the bladder is correctly segmented; however, an additional surface, which does not correspond to the bladder, was identified as bladder tissue. Figure 10g represents an ideal case with a high correlation between the ground truth and the CNN-segmentation. Finally, Figure 10h shows a case where the bladder was not found by the algorithm due to the really low contrast between the (compressed and almost empty) bladder and the surrounding tissue, as can be seen in Figure 10d. Examples of the ground truth segmentation for the cases presented above are given in Figure 10i–l.

**Figure 11:**
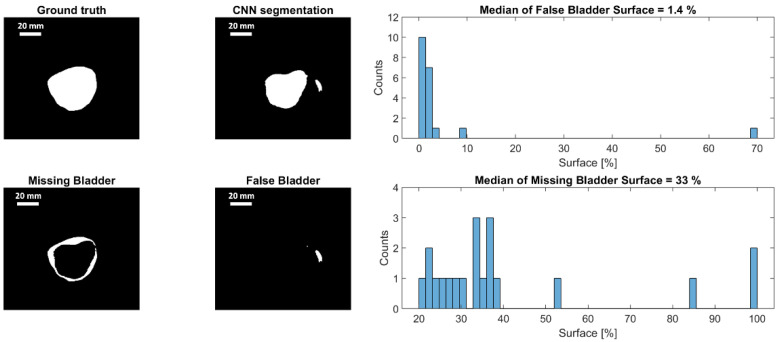
Statistical analysis of the accuracy of bladder segmentation and example of one segmentation highlighting the differences between the expert ground truth and the CNN segmentation. Areas not recognized as bladder by the CNN are marked as “missing bladder”, areas erroneously segmented as bladder by the CNN are marked as “false bladder”. The relative fractions of “missing” and “false” bladder in the different segmented data sets are shown as histogram in the right column.

For a qualitative analysis of the segmentation quality, the percentage of the bladder surface that has not been identified as bladder by the CNN was assessed. Furthermore, the image fraction that was falsely identified as bladder tissue by the algorithm was assessed as well. Both parameters are expressed in relation to the bladder surface in the ground truth segmentation. The process of automated analysis is shown in Figure 11. First, the ground truth data were binarized for easy comparison with the CNN-segmentation, which provides binarized data by default. By comparing both images, missing bladder tissue and tissue falsely identified as bladder are identified. Finally, simple pixel counting was used to quantify the missing and false bladder surface. The analysis shows that only a very small tissue fraction (corresponding to 1.4% of the bladder surface) was falsely identified as bladder tissue. On the other side, significant parts of the bladder (median of 33%) were not recognized as such by the algorithm. As can be seen in Figure 10, this is mostly the case where clutter artifacts appear, leading to low contrast between bladder tissue and the background.

## 4. Discussion

We developed a new portable low-cost ultrasound research system designed for continuous bladder imaging and characterized its (hard- and software) components in first phantom and proband experiments to assess its potential for later use in post-operative bladder monitoring. With dimensions of 18 × 12 × 3 cm^3^ and a weight of 610 g, the system is compact enough for applications where portability is required. The ultrasound probe was integrated into compact housing (diameter of 40 mm, height of 17.5 mm) and equipped with a self-adhesive foil, which allows long-term use without manual probe positioning. The system was designed, manufactured, assembled and tested in the ultrasound department of Fraunhofer IBMT. In the design process, the focus was set not only on the performance but also on cost efficiency and limiting the total material cost for the electronics to approximately €1000. The system was designed to be as flexible as possible, and therefore it provides full control to the transmit parameters and full access to the receive data pipeline, where receive and beamforming parameters can be selected and custom filters and reconstruction algorithms can be integrated into the real-time pipeline. Full data access to the receive pipeline and in particular real-time availability of the pre-beamformed channel data (up to 300 frames/s in our study) is not provided by clinical sonography systems and makes the system future-proof for other types of applications such as raw radio-frequent signal processing and ML modeling. On the other hand, most research systems are not certified for medical use. Accordingly, the combination of low-cost and the above-described flexibility makes the MoUsE system an ideal tool for research and educational purposes in ultrasound imaging. In order to ease the transfer of new ultrasound imaging approaches into clinics, the technical prerequisites such as data access must be provided and regulatory constraints must be respected as well. For this reason, we performed various tests according to safety standards for medical devices, such as electrical safety, electromagnetic compatibility and acoustic safety. Compliance to these standards was shown and the corresponding test protocols are available; this is of great value when seeking an ethics clearance for exploratory clinical studies.

In order to cover most of the clinical applications of diagnostic ultrasound, we chose a frequency range of 100 kHz–10 MHz as the target specification and validated the bandwidth in our study. The imaging performance of the MoUsE is mainly dependent on the transducer that is used. Our phased array probe with 32 elements represents a compromise between opening angle and sensitivity. A smaller pitch would have been preferred since a larger opening would have resulted, which is crucial for effective plane wave compounding. On the other hand, given the demonstrated image depth of more than 10 cm in the standard CIRS phantom and more than 15 cm in the human abdomen, imaging of the entire bladder would have been difficult to achieve with a smaller aperture size generating less acoustic energy output. The comparison of the image metrics obtained with different beamforming approaches underlines the potential of software-based reconstruction methods, and thereby, the need to have access to pre-beamformed channel data.

Having high-contrast image data is particularly needed when subsequent image processing steps are performed for automated analysis of the data, such as in our first application of bladder segmentation. We demonstrated the general functionality of our CNN for segmentation in abdominal ultrasound images. However, the analysis showed that a high contrast is crucial to prevent segmentation artifacts. This is underlined by the comparison with earlier work on the use of CNNs for bladder segmentation from ultrasound data [26,27], where a higher correlation between the automatically determined and the manually segmented bladder volumes was obtained. However, it should be mentioned, that the cited work was based on the use of high-end clinical ultrasound devices, which provide higher contrast, and two orthogonal B-mode images were acquired for obtaining quantitative values for the bladder volume [26]. The assessment of the actual bladder volume can hardly be achieved with high accuracy using single cross-sectional B-mode images, and therefore it is beyond the scope of the presented work. However, the impact of the lower SNR when compared to ultrasound data acquired with high-end clinical ultrasound machines needs to be closely investigated, particularly since the bladder cross-sectional surface was systematically underestimated. This was due to clutter signals occurring at the bladder border that were recognized as background tissue by the CNN. On the other hand, background tissue was very reliably identified with very few “false positive” areas (background tissue falsely identified as bladder).

Despite the first proof-of-concept, further investigation is needed to enhance the performance of the overall approach. In particular, the network size needs to be improved in order to allow better use on mobile devices with limited computing capabilities. Since the analysis has shown the importance of SNR for the accurate segmentation and the potential of more sophisticated beamforming approaches for contrast improvement, the optimization of image CR and CNR will be the focus of our future work. Furthermore, we will investigate if training the algorithm with more diverse data (different, and in particular, lower contrast levels) will yield higher accuracy. In summary, in applications such as the monitoring of POUR, where a significant or even dramatic and thereby potentially harmful increase in bladder volume can occur, the proposed approach provides sufficient sensitivity. However, for applications where a precise quantitative assessment of the bladder volume is needed, further enhancement of the performance is needed and will be investigated using refined beamforming approaches and improved training of the CNN.

Finally, beyond this first study on bladder monitoring, we will seek to use MoUsE and its unique combination of device mobility, flexibility and data access in other medical ultrasound applications. Although the number of transmit/receive channels is currently limited to 32, a synchronization scheme that combines several MoUsE systems for a higher total channel count is currently under development. A wireless interface is already available on the hardware, but is not implemented in software, which is also a work in progress and would allow easier use in future mobile ultrasound applications. A battery-powered version of MoUsE is in development as well. The possibility of transferring existing classification tasks using machine learning on radio-frequent data in addition to the image-based approach will also be investigated.

## Figures and Tables

**Table 1:** MoUsE system performance and features.

Dimensions	184 mm × 123 mm × 33 mm
Weight	610 g
Power consumption	12 W
Power supply	12 V DC, medical certified power supply, lithium-ion battery packs for future fully mobile applications
Transmitter	32 channels, Tri-state pulser, max voltage ± 100 V
Receiver	32 channels Bandwidth: 100 kHz–10 MHz Gain: up to 44.3 dB Up to 50 MHz sampling rate with a resolution of 12 Bit per sample
Interface	USB 3.0
RAM	8 GBit internal RAM
Imaging	Plane wave compounding, custom algorithms can be implemented
Software	Clinical type user interface USPilot, SDK for programming system from 3rd party applications in C#/C++/Matlab
Transducer specifications	32 elements Pitch = 500 µm Centre F\frequency = 3 MHz
